# Commuting in metapopulation epidemic modeling

**DOI:** 10.1038/s41598-021-94672-w

**Published:** 2021-07-26

**Authors:** Azi Lipshtat, Roger Alimi, Yochai Ben-Horin

**Affiliations:** Soreq Nuclear Research Center, Yavne, 81800 Israel

**Keywords:** Computational science, Epidemiology

## Abstract

The COVID-19 pandemic led authorities all over the world to imposing travel restrictions both on a national and on an international scale. Understanding the effect of such restrictions requires analysis of the role of commuting and calls for a metapopulation modeling that incorporates both local, intra-community infection and population exchange between different locations. Standard metapopulation models are formulated as markovian processes, and as such they do not label individuals according to their original location. However, commuting from home to work and backwards (reverse commuting) is the main pattern of transportation. Thus, it is important to be able to accurately model the effect of commuting on epidemic spreading. In this study we develop a methodology for modeling bidirectional commuting of individuals, without keeping track of each individual separately and with no need of proliferation of number of compartments beyond those defined by the epidemiologic model. We demonstrate the method using a city map of the state of Israel. The presented algorithm does not require any special computation resources and it may serve as a basis for intervention strategy examination in various levels of complication and resolution. We show how to incorporate an epidemiological model into a metapopulation commuting scheme while preserving the internal logic of the epidemiological modeling. The method is general and independent on the details of the epidemiological model under consideration.

## Introduction

The outbreak of the COVID-19 pandemic has inspired developing, simulating and analyzing of numerous computational epidemiologic models (EMs)^1–4^. The goal of these models is understanding the epidemic dynamics and providing reliable predictions about its expected spread in time and space, on either a national or a global scale. The vast majority of these models falls within the class of ‘compartmental models’^5–7^. In this type of models, the entire population is divided into several compartments, such as healthy individuals, infected, sick, dead, recovered etc. The time dependent size of the compartments is governed by a set of coupled ordinary differential equations (ODEs). The models differ from each other by the number of compartments and the corresponding clinical situation they represent.

A common underlying assumption in many of these models is the homogeneity of population^5,7^. Namely, it is assumed that any two individuals have the same chance to meet each other. This is a good description of a local epidemic spread which is fully justified as long as infection within a single community is being concerned. This assumption enables formulating the model as a set of mean field equations. In recent years network models have been proposed, which do not assume homogeneous mixing^8–11^. Under conditions of a well defined group, either with homogeneous or heterogeneous mixing, one may average all these meetings into clumped contagion rate coefficients and formulate an ODEs model. However, on larger scales, such a population averaging does not hold any more. On national and international scales the disease is being transferred by individuals who commute from one location to another, and play the role of an infection seed in their new location. This is a different mechanism, whose effect cannot be incorporated into the standard compartmental models^12^.

Metapopulation models are aimed at modeling the travel effect. These models assume local populations connected by migrating individuals. In epidemiological contexts, a network of locations (a.k.a ‘patches’) is defined, with a transfer matrix that defines the interaction between them. Then a compartmental model is employed for each location^13,14^. Most of these models assume unidirectional travel and are tailored for global spreading of diseases. This class of models describes well propagation and transmission on a global scale.

Various types of models may be found within the class of metapopulation models. Models may be either stochastic or deterministic. They may differ also with respect to the resolution at which the model is formulated. Some models follow only the population size in each location. In these cases transfer is implemented by an appropriate decrease of population size in one patch and respective increase at another one. Other models are more detailed and follow the location of each individual. Such models may encompass important aspects which cannot be observed by the incorporated models, such as routine trajectories of certain individuals or a ‘super spreader’—a single person who visits multiple locations. The main disadvantage of the detailed models is their computational cost. These models require large computation resources, they are cpu time consumers, and thus are limited in population size which can be efficiently modeled.

On a national scale, the main traveling is performed in context of work commuting. Such a traveling pattern is known to have different characteristics and spreading effects than the random unidirectional travel^15^. The reason for that is that commuting takes place on a periodic pattern with same people commuting to the same place, as opposed to the random pattern of the global traveling. Reverse commuting is a pattern in which each individual spends most of the day in one location (considered to be the residence city) and several hours in another (working location). This is an important aspect of commuting. Ignoring the reverse commuting and taking into account unidirectional commuting, may cause misleading results. For example, under conditions of high commuting frequency, relative to contagion rate, infected individuals would diffuse to all patches, and standard unidirectional metapopulation models would approach a steady state of same morbidity everywhere, regardless of the initial spatial distribution. Accurate commuting is expected to return each individual to her original location, which would significantly slow down the spread of disease.

The reason for not including reverse commuting in many models is the difficulty in calculating the clinical state of the commuting individuals at the end of the working day. If a group of $N_{ij}$ individuals commutes from location *i* to *j*, with fraction $f_{ij}=I_{ij}/N_{ij}$ of infected ones, this fraction at the end of the day is neither the original fraction nor the fraction in the general population presented in *j* at that time. In cases where reverse commuting is taken into account, the computational scheme is based on separate simulation for each commuting group, such that for a metapopulation model of *M* locations there will be $M^2$ interacting epidemic models^16,17^.

In this study we introduce an efficient algorithm for commuting in metapopulation models, which does not require detailed formulation to the level of individuals or any proliferation of epidemic models beyond the number of patches. The algorithm is demonstrated in the context of integrated metapopulation EM for the state of Israel.

## Methodology

**Figure 1:**
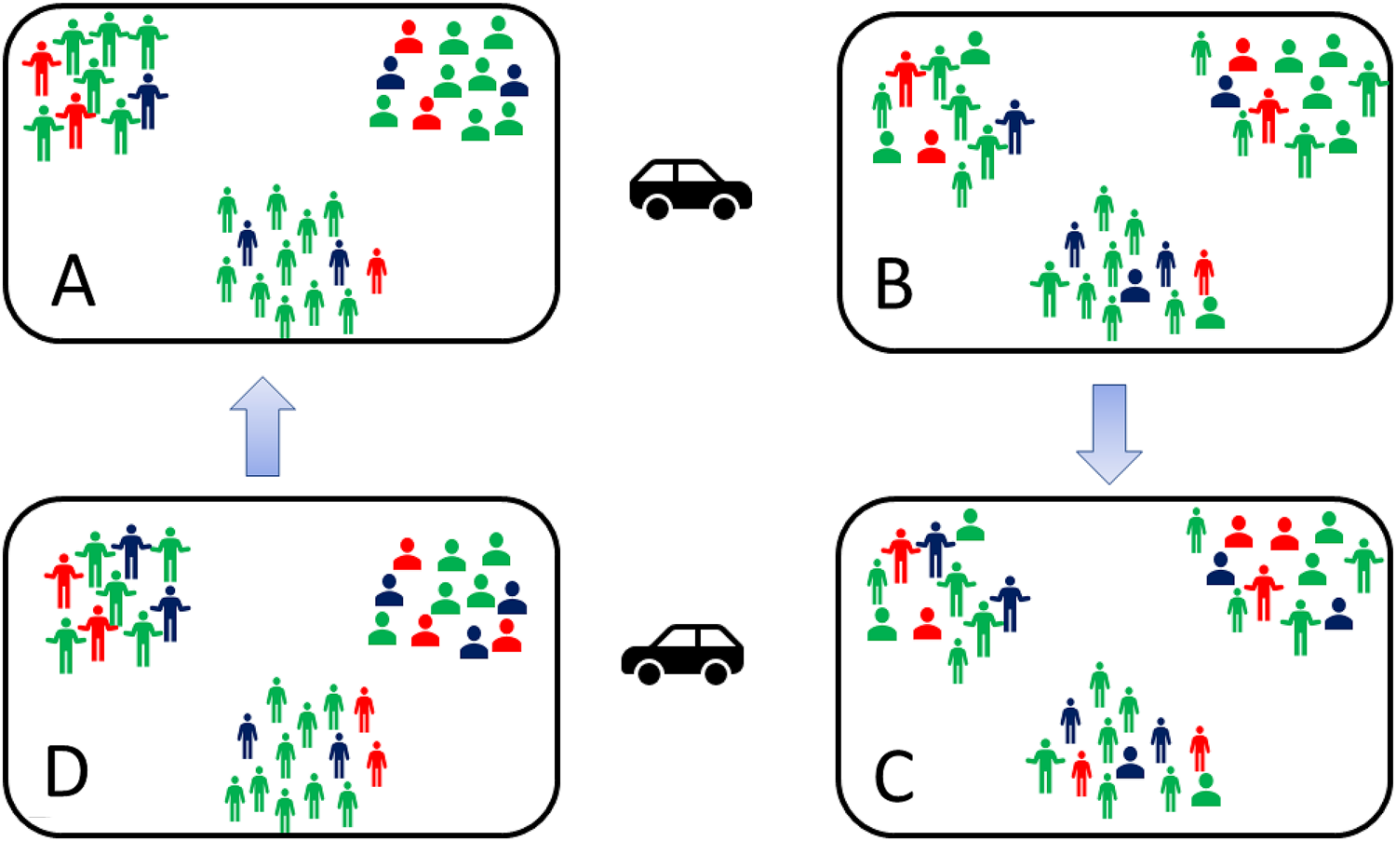
Conceptual view of the model. Different colors represent different epidemic conditions e.g. uninfected, sick etc., and different icons represent the residents of different locations. (**A**) The situation at the beginning of the day. (**B**) After transportation, the number of people in each location has changed, but total number in each compartment is conserved. (**C**) Due to the epidemic model individual may move to a different compartment. No change in total number at each location. (**D**) Reverse commuting. Number in each location is the same as in the morning. Total number in each compartment is the same as in (**C**).

The metapopulation approach is inspired from graph theory network in which communicating sites are modeled as nodes and the connections between the sites are modeled as links or edges between the nodes. In each single node the population evolution follows a given epidemiologic behavior, which can be as simple as a basic SIR model^5^ or a more sophisticated one. In our example transportation between all sites takes place twice a day. The first time is at 8 A.M. when a given percentage of people moves from their home to other cities and working center, and the second time is when the same number of individuals returns back to their living places after 8 h (at 4 P.M.) (Fig. 1). The number of individuals moving from one place to the other is determined by a mixing matrix *A*, where the $A_{ij}$ element being the fraction of population moving from site *i* to site *j*. The diagonal entries $A_{ii}$ contain the population fraction which do not commute, such that for each location *i* by definition $\sum _j A_{ij} = 1$.

It should be noted that the epidemic model runs independently for each location. Thus, one can use a separate model for each place. In particular, one can adjust a unique set of parameters for any particular location, based on demographic or behavioral differences between different communities^18^. This feature is useful when considering a heterogeneous population, as is it the case in Israel. The total number of equations to be solved is a product of number of locations considered by the number of compartments in the local compartmental epidemic model. It can run easily on any standard laptop computer and thus this model serves as an efficient tool that can be considered as a good compromise between the detailed description as in heavy stochastic models, and the ease of use and flexibility of simple ODEs models.

### The bidirectional commuting scheme

In order to illustrate the scheme we assume that the entire population *P* is divided into subpopulations (compartments) according to their clinical situation (e.g. uninfected, sick etc.). We denote the subpopulations by $\{{{{\mathscr {P}}}}_k\}$, $k=0,1,\ldots N-1$. The number *N* and identity of subpopulations are defined by the epidemic model. The size of the subpopulation ${{{\mathscr {P}}}}_k$ in site *i* is denoted by $[{{\mathscr {P}}}_k]_i$. Some of these subpopulations may commute and some do not. For the sake of clarity, we describe the population transfer mechanism for a sequential epidemiological model, i.e. individuals from ${{\mathscr {P}}}_k$ can change their clinical situation and move only to ${{\mathscr {P}}}_{k+1}$. We will later show how to generalize it for a non-sequential model, where individuals from one subpopulation may move to- or arrive from multiple other subpopulations.

Before the morning commuting step we keep in memory the population of each group in each city, $\{{{\mathscr {P}}}_k^{(0)}\}$. (Superscript indices denote step or time). At 8 A.M. a fraction of the population commutes from one site to another using the matrix *A*. The total number of people from subpopulation ${{\mathscr {P}}}_k$ that have commuted from place *i* to place *j* is denoted by the matrix $D_{k}$, where

1 $$\begin{aligned} D_{k}(i,j)=A_{ij}\times [{{\mathscr {P}}}_k]_i . \end{aligned}$$

Note that for generality, the matrix *A* may be unique for each compartment, i.e. $A=A(k)$ without affecting the scheme. In particular, for non-commuting compartments $A=0$. For readability we use the same matrix for all compartments. The population number is updated after the mixing and can be written as:

2 $$\begin{aligned} {[}{{\mathscr {P}}}_k]_i^{(1)}= [{{\mathscr {P}}}_k]_i^{(0)}+\sum _j\left[ {D_{k}(j,i) - D_{k}(i,j)}\right] \end{aligned}$$

Similar terms are computed for all other commutable subpopulations. Then the epidemic model runs again (locally) during an 8 h “working day”. In each place *i*, at the end of the working day, the size of each subpopulation, e.g. $[{{\mathscr {P}}}_k]_i^{(1)}$ has evolved to $[{{\mathscr {P}}}_k]_i^{(2)}$. Then at 4 P.M. the population is updated to $[{{\mathscr {P}}}_k]_i^{(3)}$ due to returning of all workers back to their home locations. An individual who started the working day (time (1)) at subpopulation ${{\mathscr {P}}}_k$ may remain at the end of the day (time (2)) at the same subpopulation, or move to the next one, ${{\mathscr {P}}}_{k+1}$. We assume that within a single working day one cannot move between compartments more than once. Thus, there is a fraction $\phi _{k,i}$ of those individuals who remained in ${{\mathscr {P}}}_k$ in site *i*. The rest, namely $(1-\phi _{k,i})$, moved to ${{\mathscr {P}}}_{k+1}$. Accordingly, $\phi _{k,j}D_k(i,j)$ should return to subpopulation ${{\mathscr {P}}}_k$ in site *i*, and $(1-\phi _{k,j})D_k(i,j)$ will return to subpopulation ${{\mathscr {P}}}_{k+1}$. Thus, if subpopulation ${{\mathscr {P}}}_k$ may contribute to ${{\mathscr {P}}}_{k+1}$ and receive individuals from ${{\mathscr {P}}}_{k-1}$, the subpopulation size after commuting back is given by

3 $$\begin{aligned} {[}{{\mathscr {P}}}_k]_i^{(3)}= [{{\mathscr {P}}}_k]_i^{(2)}+ & {} \sum _j [ \phi _{k,j} D_k(i,j) + ( 1-\phi _{k-1,j})D_{k-1}(i,j) \,]\nonumber \\- & {} \sum _j [ \phi _{k,i} D_k(j,i) + (1-\phi _{k-1,i})D_{k-1}(j,i) \,] \end{aligned}$$

The first sum at Eq. (3) represents the contribution of individuals who reside in site *i* and commuted to *j*, and the second sum is for those who spent the working day at *i* but live in *j*. In each sum, the first term is the number of those who started the day at subpopulation ${{\mathscr {P}}}_k$ and remained in the same subpopulation, and the second term is the number of those who started the day in subpopulation ${{\mathscr {P}}}_{k-1}$ and changed their clinical state such that they are now in ${{\mathscr {P}}}_k$. Obviously for $k=0$ the terms that refer to ${{\mathscr {P}}}_{k-1}$ should be omitted.

Note that all our matrices include non-zero diagonal elements; therefore the same equations apply also for people that have stayed in their place during working day. Once all populations have been updated, a new cycle can start again by running 16 h of EM from 4 P.M. to 8 A.M. the next day.

In the following we describe how to find recursively the fractions $\phi$: We start with subpopulation ${{\mathscr {P}}}_0$ which is an initial clinical state, i.e. there is no other subpopulation from which one can move to ${{\mathscr {P}}}_0$. Typically this is the uninfected subpopulation. The difference $\Delta _0 = [{{\mathscr {P}}}_0]^{(1)} - [{{\mathscr {P}}}_0]^{(2)}$ is the number of individuals who moved from subpopulation ${{\mathscr {P}}}_0$ to ${{\mathscr {P}}}_1$. (For simplicity we omit the subscript *i* as we refer to a single location). By definition $\Delta _0 \ge 0$. The fraction of individuals who remained in ${{\mathscr {P}}}_0$ is $\phi _0 = [{{\mathscr {P}}}_0]^{(2)} / [{{\mathscr {P}}}_0]^{(1)}$. The rest moved to ${{\mathscr {P}}}_1$.

Now we turn to the next subpopulation, ${{\mathscr {P}}}_1$. At the end of the working day its size was $[{{\mathscr {P}}}_1]^{(2)}$, out of which $\Delta _0$ are “new comers”, i.e. individuals who started the day in subpopulation ${{\mathscr {P}}}_0$. Thus, the fraction of those who started at ${{\mathscr {P}}}_1$ and didn’t move to another subpopulation is

$$\begin{aligned} \phi _1 = \frac{[{{\mathscr {P}}}_1]^{(2)} - \Delta _0}{[{{\mathscr {P}}}_1]^{(1)}}. \end{aligned}$$

The number of people who moved from ${{\mathscr {P}}}_1$ to ${{\mathscr {P}}}_2$ is $\Delta _1 = [{{\mathscr {P}}}_1]^{(1)} - \left( [{{\mathscr {P}}}_1]^{(2)}-\Delta _0\right)$. In general, if a subpopulation ${{\mathscr {P}}}_k$ received $\Delta _{k-1}$ individuals during a working day, the size of its contribution to ${{\mathscr {P}}}_{k+1}$ is given by

4 $$\begin{aligned} \Delta _k = [{{\mathscr {P}}}_k]^{(1)} - \left( [{{\mathscr {P}}}_k]^{(2)}-\Delta _{k-1}\right) , \end{aligned}$$

and the fraction of those who remained in the subpopulation is

5 $$\begin{aligned} \phi _k = \frac{[{{\mathscr {P}}}_k]^{(2)} - \Delta _{k-1}}{[{{\mathscr {P}}}_k]^{(1)}} \end{aligned}$$

Note that this scheme meets all logical requirements: For terminal subpopulations such as ‘dead’ or ‘recovered’, since ${{\mathscr {P}}}_k^{(2)} = {{\mathscr {P}}}_k^{(1)} + \Delta _{k-1}$, we get $\phi _k=1$ as expected. If no infection takes place, then by definition $\Delta =0$ and all commuters return to their original subpopulation. This is in contrast to standard metapopulation models, where traveling *per se* may change the morbidity rate in some locations, even without any infection taking place. Furthermore, even though we follow the number of commuters, but not individuals, yet in this scheme it is not possible for any individual to “go back” in the EM, i.e. it cannot be that more uninfected ones return than the number that left the city in the morning.

It should also be noted that in the presented scheme, the morning commuters from *i* to *j* are taken from the local epidemic distribution of patch *i*, which is not necessarily the same as the distribution among those individuals who returned from *j* in previous day. In some cases we would be interested in keeping the same individuals going on the same route every day. This can be done by using the same methodology as it is done when following the returning commuters at the end of a working day.

### Non sequential models

We have introduced the calculation of the fractions $\phi$ for sequential EMs of the form ${{\mathscr {P}}}_{k-1} \rightarrow {{\mathscr {P}}}_k \rightarrow {{\mathscr {P}}}_{k+1}$. Generalization of the computation for non sequential models is easily done. In case the model enables moving to subpopulation ${{\mathscr {P}}}_k$ from several subpopulations, respective terms similar to the second term of each sum in Eq. (3) should be added for each such subpopulation. In other words, instead of considering only those individuals who moved from ${{\mathscr {P}}}_{k-1}$, one should take into account those individuals who came from all possible compartments.

$1-\phi _k$ is the fraction that moved from ${{\mathscr {P}}}_k$ to ${{\mathscr {P}}}_{k+1}$. In cases where the EM enables transfer from one subpopulation to multiple other subpopulation, the transfer fraction $1-\phi$ in Eq. (3) should be distributed among these subpopulations with the same ratio as their respective kinetic rates. For example: consider a model in which a sick (*S*) person can either recover (*R*) with rate $\alpha$, or die (*D*) with rate $\beta$. Then in terms relating to $1-\phi _S$ in Eq. (3) should be replaced by $\frac{\alpha }{\alpha +\beta }(1-\phi _S)$ in the equation for $[{{\mathscr {P}}}_R]$ and by $\frac{\beta }{\alpha +\beta }(1-\phi _S)$ in the equation for $[{{\mathscr {P}}}_D]$. In the next section we provide an example for such a non-sequential model.

## Examples and applications

### The epidemiological model

**Figure 2:**
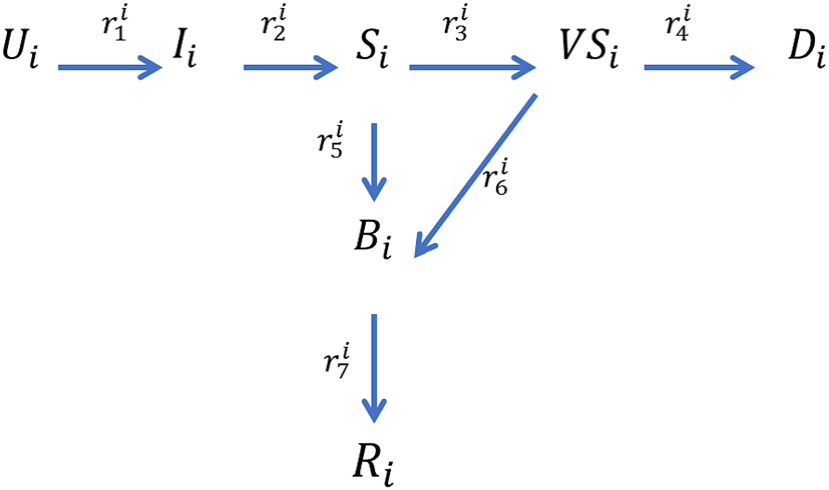
A schematic view of the extended SEIR EM, adapted from Ref.^4^.

We now very briefly summarize the formalism of the extended SEIR epidemic model we have used. This model was specifically designed and calibrated for the COVID-19 pandemic^4^. In this model seven mutually exclusive subpopulations are being considered. The subpopulations are: uninfected (U), infected in the incubation period (I), sick (S), very sick (VS), dead (D), better (B) and recovered (R). As opposed to the well known SIR model, this model is not sequential, i.e. there is a compartment which may contribute to two other compartments (‘Sick’ may become either “Very Sick” or “Better”) and a compartment which receives individuals from two subpopulations, as shown in Fig. 2.

The governing equations are:

6 $$\begin{aligned} \frac{dU}{dt}= & {} -\left( k_{11}I + k_{12}S +k_{13}VS +k_{14}B \right) \frac{U}{P}\nonumber \\ \frac{dI}{dt}= & {} \left( k_{11}I + k_{12}S +k_{13}VS +k_{14}B \right) \frac{U}{P}-k_2I\nonumber \\ \frac{dS}{dt}= & {} k_2I-k_3S-k_5S\nonumber \\ \frac{dVS}{dt}= & {} k_3S-k_4VS-k_6VS \nonumber \\ \frac{dD}{dt}= & {} k_4VS \nonumber \\ \frac{dB}{dt}= & {} k_5S+k_6VS-k_7B\nonumber \\ \frac{dR}{dt}= & {} k_7B \end{aligned}$$

For simplicity, in order to minimize the number of parameters, we follow DeVisscher and take $k_{12}=k_{11}/2$, $k_{13}=k_{11}/3$, and $k_{14}=k_{11}/4$^4^. In order to compare the results of this model to the standard SIR model, we have grouped all sick populations in the extended model into one single population. Similar grouping was done for the *R* population. The grouping took place as a post processing step, after completion of the calculation, and is done for presentation purpose only. This equivalence is summarized in Table 1.

**Table 1:** Equivalence of the two epidemiology models.

SIR model	extended SEIR model
S	U
I	I+S+VS+B
R	D+R

The epidemic model have been incorporated into a metapopulation model. This model includes 55 Israeli cities which include about $60\%$ of the population in Israel. In the online Supplementary Information we provide more details about the metapopulation model and present results of using it with both the SIR and the extended SEIR models.

### Effect of travel limitation

Travel limitation is a common step taken by authorities in various countries. Its goal is both preventing transmission of the virus out of an infected area, and protecting uninfected areas from arrival of the disease. However, regional travel limitations which prevent inter-city transportation cannot challenge intra-community infecting. As a consequence, its main effect is delaying the outbreak, but it cannot eradicate it.

Albeit the travel limitation may be considered as significant, one should keep in mind that a complete and definite travel limitation is practically unfeasible. What should be the effect of partial travel limitation? We have simulated such a limitation by scaling down all transportation by a large factor, namely by dividing the transportation matrix by 10, 100, or 1000. Although one may consider different levels of travel limitation in different regions, for simplicity we used the same reduction factor for all commuting routes.

**Figure 3:**
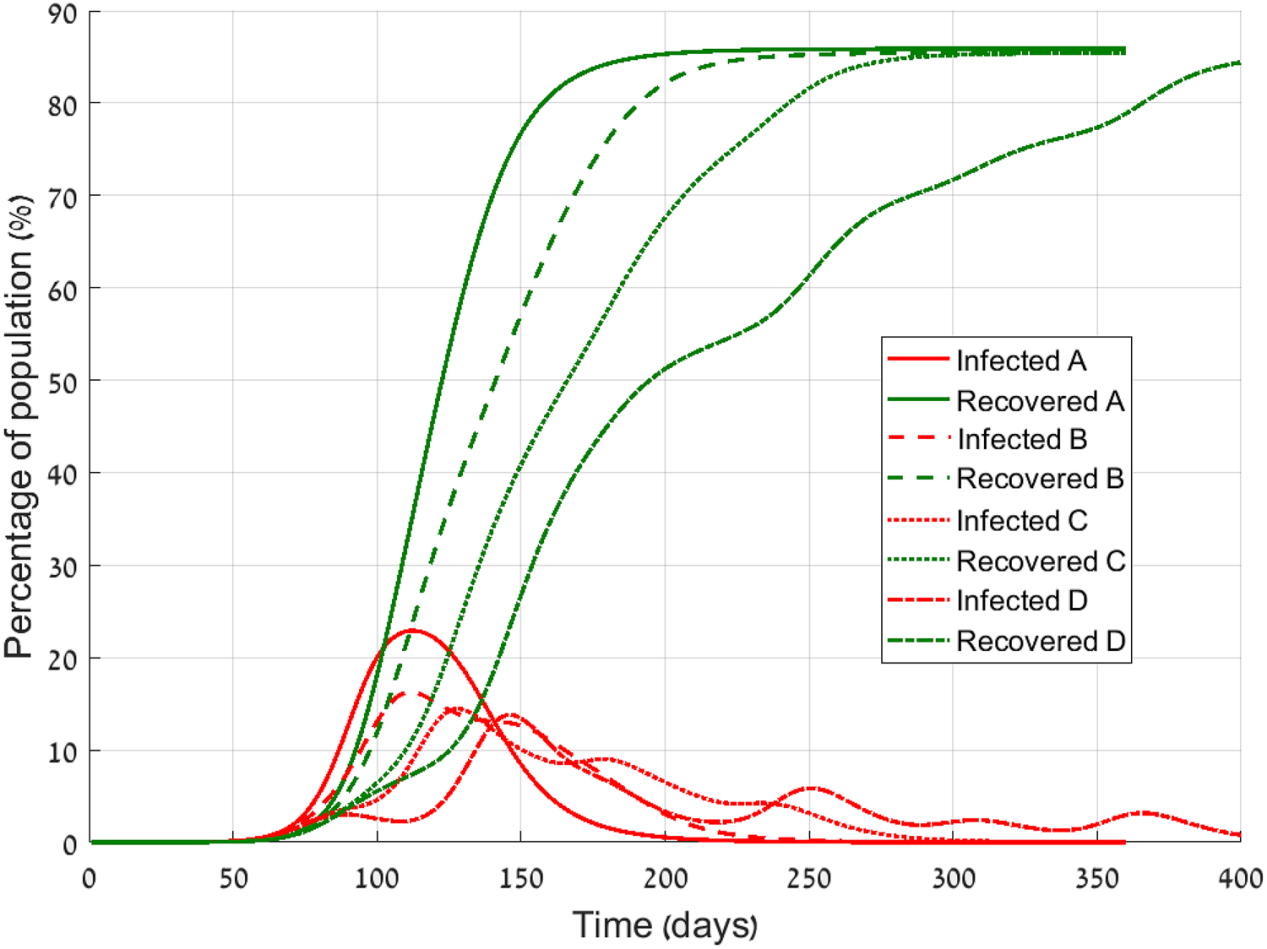
Morbidity and recovery with partial travel limitation starting at day 1 (DeVisscher model). A—standard commuting, B—90% travel limitation, C—99% travel limitation, D—99.9% travel limitation. Subpopulations are grouped according to Table 1 in order to make presentation equivalent to SIR model.

The results presented in Fig. 3 show the total number of sick people and recovered ones. The sick group integrates all sickness levels, and the recovered includes dead as well, as explained in Table 1. We present the results for four scenarios: standard commuting, reduction of 90% in commuting due to travel limitations, 99%, and 99.9% reduction of the transportation. All travel limitation policies were imposed from the very beginning. In all cases the final number of recovered individuals is almost the same, i.e. the same number of people had experienced the disease at some time. Even reduction in three orders of magnitude made a decrease of only 1/3 in the maximal number of coexisting patients, but not in the total number. Reduction in commuting spans the outbreak over a longer period, but at the end of the day the outbreak stops due to herd immunity. As long as no other intervention steps are taken, a vast majority of the population would experience the disease sooner or later. The differential delay is clearly seen in the maximal reduction case (*D*), where local outbreaks at different times lead to ripples in the sick graph. Similar results were obtained by the SIR model.

### Effect of social distancing

Since viral transmission highly depends on how close to each other people stand, one of the first steps taken is social distancing. Reducing the number of mutual meetings can be expressed as a reduction in infection rate. Computationally it is implemented by lowering the value of the infection rate constant ($k_{11}$ in the extended SEIR model).

**Figure 4:**
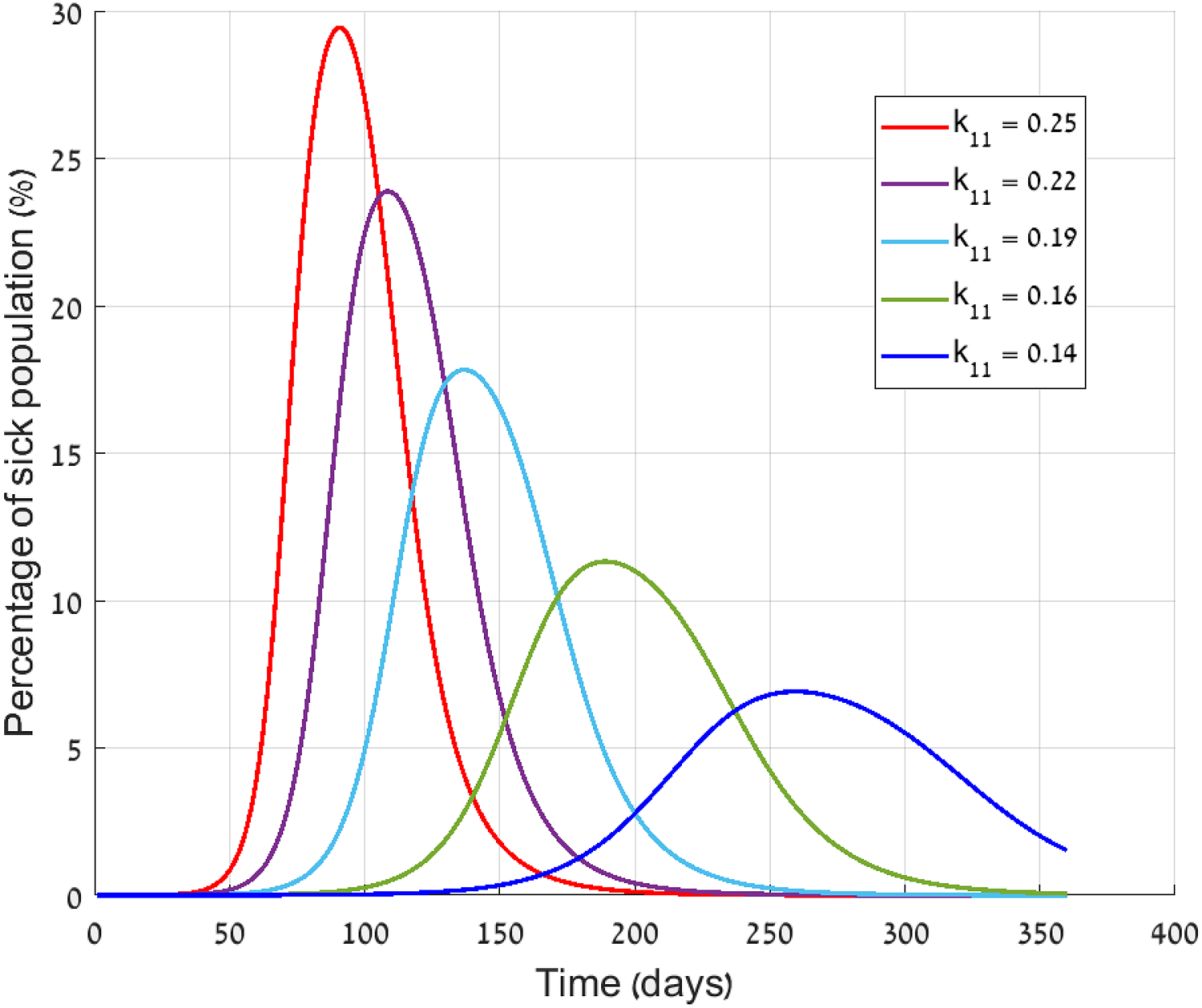
Morbidity for various values of $k_{11}$ (DeVisscher model).

In Fig. 4 we present the expected number of sick people for various values of $k_{11}$. Beyond considering a range of societal behavior, no direct intervention steps were taken, so herd immunity is responsible to the outbreak termination. It is shown that a change of factor smaller than 2 is sufficient to reduce the peak value by more than 50%. This effect is by far more significant than the travel limitation effect.

### Comparison with the naïve commuting algorithm

**Figure 5:**
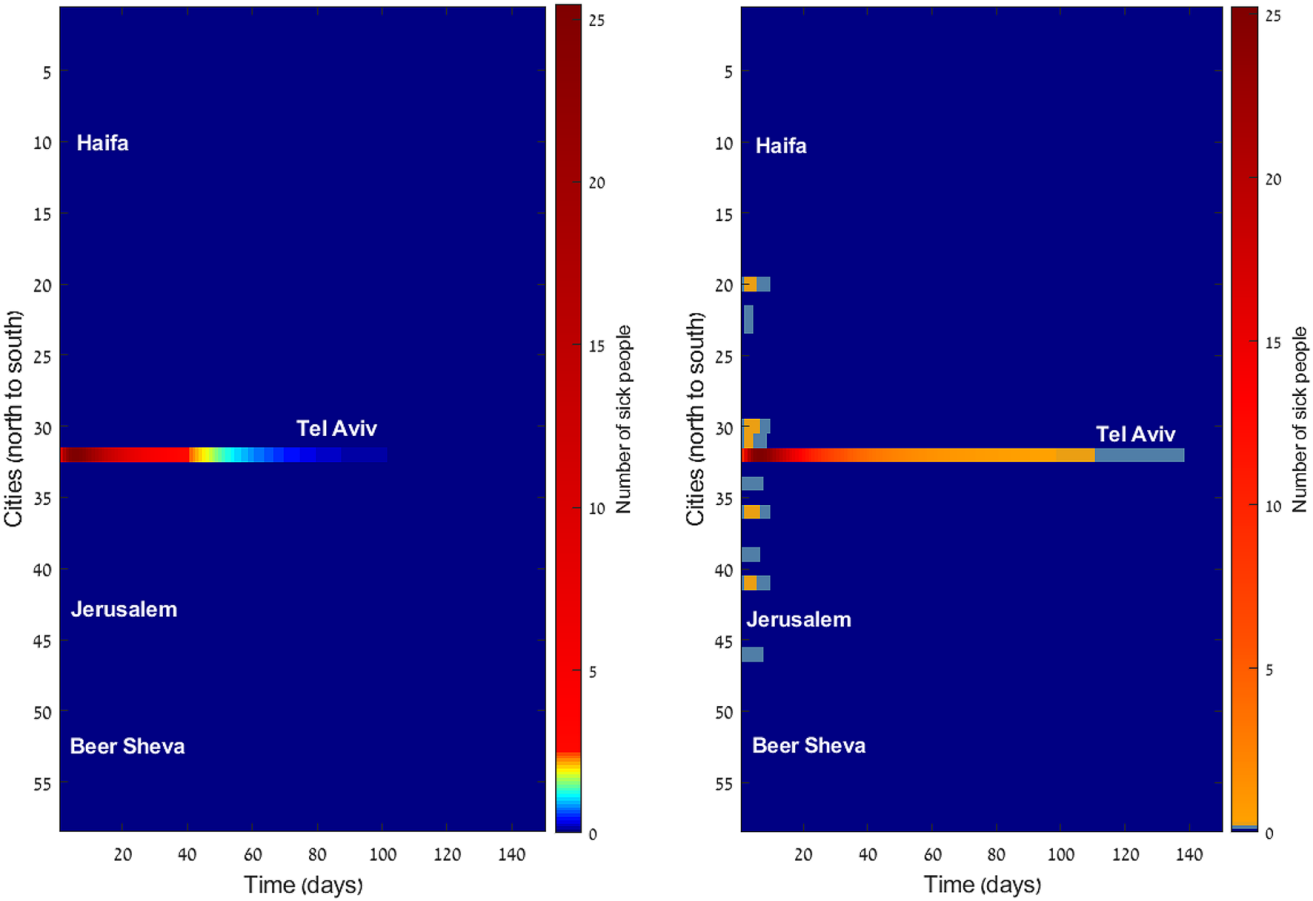
Number of sick individuals in 55 locations (sorted from north to south). Infection rate is zero. Left: the accurate reverse commuting method, morbidity is limited to Tel-Aviv. Right: naïve approach, morbidity incorrectly diffuses to other locations.

To demonstrate the importance of accurate computation of the bidirectional commuting, we compare our algorithm to the naïve method. First, we consider reverse commuting taken from the local distribution. If a group of $N_{ij}$ individuals commutes from location *i* to *j*, with fraction $f_{ij}=I_{ij}/N_{ij}$ of infected ones, then at the end of the working day, the same total number of individuals returns back to *j*, with the infected fraction which is the same as the fraction of infected ones out of the general population in *j*. Then, we use the reverse commuting scheme presented here. The two options were simulated for a range of parameters. As expected, under conditions where the main contagion thread is the intra-communal meeting (i.e. high infection rate and low commuting rate), the details of the reverse commuting method have no significant effect, and the two methods present similar results. However, as the role of commuting increases, differences start to emerge. In Fig. 5 we present the extreme case of no contagion, i.e. infection rate is zero and the disease spreads out only by commuting. We start with a seed of 100 infected individuals in Tel-Aviv. The naïve approach of determining the fraction of returning infected individuals based on local morbidity, exhibits diffusion of infected individuals to many locations. Our approach, on the other hand, sends back correctly all the infected ones back to Tel-Aviv and keeps all other locations free of disease. These results are independent on the details of the epidemic model. We have observed similar results for other models, such as SIR.

## Summary and conclusions

In this study we have developed a computational scheme for metapopulation EMs which is capable of simulating bidirectional commuting. This type of modeling may serve as an important tool for policy makers when considering various intervention steps. For example, it is shown that social distancing is much more effective than travel limitations. It is also shown that incorrect calculating of reverse commuting may affect the resulting dynamics and produce misleading results. The presented scheme may be easily included in any metapopulation model, and is not limited to any specific EM. Furthermore, since the EM is calculated independently for each city, one may change model parameters between different locations. As a consequence, unique characteristics of different populations may be incorporated into the metapopulation model. An example of adjusting parameters in a location-dependent manner based on real data is presented in the online Supplementary Information. The computational scheme can be further extended by considering compartment-dependent transfer matrix. Age dependent dynamics may be also included by considering each age group as a separate compartment, with the computational cost of having more compartments. This approach compromises between the detailed description at the level of individuals, as in heavy stochastic models, and the ease of use and flexibility of simple ODEs models. As such, it cannot predict the effect of unique individuals, such as super spreaders. Note also that the computational scheme requires the existence of an initial compartment ${{\mathscr {P}}}_0$. It is not applicable for circular EMs such as SIS^19^.

The presented scheme does not require more equations than the total number of compartments in all patches, as defined by the EM. Thus it is an efficient and easily implementable scheme. No special computation resources are needed in order to get accurate and quick results.

## Supplementary Information

Supplementary Information.

## Data Availability

No datasets were generated or analysed during the current study.
