# Supplementary material for: Commuting in metapopulation epidemic modeling
Source: Sci Rep. 2021 Jul 26;11:15198. doi: 10.1038/s41598-021-94672-w (PMC8313540; doi:10.1038/s41598-021-94672-w)
Supplement: Supplementary file 1 — Supplementary Information. [file 41598_2021_94672_MOESM1_ESM.pdf]

Commuting in Metapopulation Epidemic Modeling: Supplementary Information

[1], **Azi Lipshtat**[1,*], **Roger Alimi**[1], **and Yochai Ben-Horin**[1]

[1]Soreq Nuclear Research Center, Yavne 81800 Israel
[*]Eliezerli@soreq.gov.il

ABSTRACT

In the following we provide a detailed description of the transportation model used in the main paper. In addition we show some simulations and application of the metapopulation model.

Metapopulation Model

Transportation model

We use a data base of 55 Israeli cities (Figure S1 and Table S1). The list comprises not only all large to medium urban centers but also several small cities or villages located all over the country. In the present work we have used information regarding geographic location and total population in each city[3]. Further data such as age distribution of the population can be easily incorporated into the model. However, in this first approximation the results we will present do not take into account the age distribution. In the present study, in addition to regular cities, three working centers were added. The uniqueness of these places being that they are occupied only during working hours, namely from 8 AM to 4 PM.

Transportation between all sites takes place twice a day. The first time is at 8 AM when a given percentage of people moves from their home to other cities and working center, and the second time is when the same amount returns back to their living places after 8 hours (at 4PM). The number of individuals moving from one place to the other is determined by a mixing matrix A, where the A_{ij} element being the fraction of population moving from site i to site j. The diagonal entries A_{ii} contain the population fraction which do not commute, such that by definition $\sum_j A_{ij} = 1$. To demonstrate the method, we have used a gravity model based on the following assumptions for the construction of the mixing matrix:

- Four cities are defined as central cites: Tel-Aviv, Haifa, Jerusalem and Beer- Sheva. 10% of the population of any other city is traveling to the nearest central city.
- The three working hubs are defined the same way as the central cities. 5% of the population of any city is working at the nearest central hub.
- An additional 5% of the population of any city is moving to cities located up to 15 km away.
- An additional 1% of the population of any city is traveling to cities located in a radius between 15 km and 50 km.

Other sets of criteria were examined as a basis for the matrix construction. Despite some minor quantitative changes, the qualitative results and conclusions remained the same. On a general model, one may define a more complicated matrix. There could be a separate matrix for each sub-population, based on age, clinical state etc. To make it even more realistic, this synthetic transportation matrix can be tuned and adjusted for each city, based on data sources and on cultural, sociological, and demographic characteristics. As our goal in this study is to prove the concept rather than providing accurate predictions, we have used this simple version without modifications. The matrix was changed only in cases where we wanted to examine local effects.

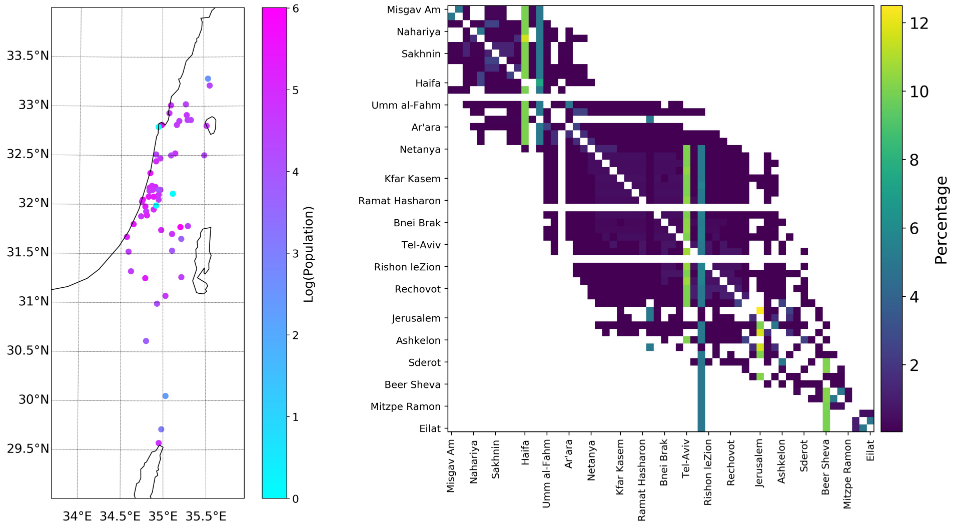

Figure S1. Left: A map of Israel with the 55 selected locations. Color denotes log of population size. (Produced using Matplotlib package[1] and python 2.7.3[2], URL: https://www.python.org/downloads/release/python-273/.) Right: The transportation matrix. The diagonal entries were removed in order to show the variation of the non-diagonal values. White color indicates no commuting ($A_{ij} = 0$).

List of cities

Table S1. List of the 55 cities and 3 industrial zones

City	Population	lat	lon
Misgav Am	339	33.28	35.55
Kiryat Shemona	24242	33.21	35.57
Maalot Tarshiha	24950	33.02	35.28
Nahariya	67565	33.01	35.1
Acco	56725	32.93	35.08
Karmiel	53838	32.91	35.29
Arraba	26687	32.86	35.34
Sakhnin	33083	32.86	35.3
Tamra	34696	32.85	35.2
Haifa	324648	32.81	34.99
Shefaram	41993	32.81	35.17
Tiberias	49378	32.8	35.53
Matam (Ind. Zone)	1	32.79	34.95
Umm al-Fahm	56860	32.52	35.15
Or Akiva	21880	32.51	34.92
Ar'ara	20363	32.5	35.1
Beit She'an	20524	32.5	35.5
Pardes Hanna Karkur	45157	32.47	34.97
Hadera	105058	32.44	34.92
Natania	252898	32.32	34.85
Ra'anana	90095	32.19	34.87
Kfar Saba	110743	32.18	34.91
Herzliya	109847	32.17	34.84
Hod Hasahron	65870	32.15	34.88
Jaljulia	10591	32.15	34.95
Kfar Kassem	24575	32.15	34.97
Ramat Hasharon	51853	32.14	34.84

Table S1 - continuation

City	Population	lat	lon
Barkan (Ind. Zone)	1	32.11	35.12
Rosh Ha'ayin	64205	32.1	34.95
Bnei Brak	212140	32.08	34.83
Petah Tikva	271532	32.08	34.89
El'ad	48455	32.05	34.95
Tel-Aviv	554208	32.05	34.76
Bat Yam	158499	32.03	34.75
Airport City (Ind. Zone)	1	31.99	34.92
Rishon leZion	271798	31.98	34.79
Lod	84021	31.95	34.89
Ness Ziona	49549	31.93	34.8
Rehovot	154475	31.89	34.81
Yavne	50006	31.88	34.74
Ashdod	254589	31.8	34.65
Ma'ale Adumim	41223	31.78	35.3
Jerusalem	1012096	31.77	35.21
Beit Shemesh	134677	31.74	34.98
Beitar Illit	63051	31.7	35.11
Ashkelon	158099	31.67	34.57
Tekoa	4382	31.65	35.22
Kiryat Arba	8024	31.53	35.11
Sderot	29562	31.52	34.59
Ofaqim	33922	31.32	34.62
Arad	31344	31.26	35.22
Be'er Sheva	221699	31.25	34.79
Dimmona	39678	31.07	35.03
Yeruham	11362	30.99	34.93
Mitzpe Ramon	5599	30.61	34.8
Be'er Ora	232	30.05	35.03
Neot Semadar	1062	29.71	34.98
Eilat	67518	29.57	34.95

Comparison between the two epidemiological models

Our reference scenario is an outbreak starting from a seed of 10 individuals in Tel-Aviv. This initial condition was taken because Tel-Aviv is a large city that attracts many tourists. In this scenario, no intervention is taken at any time. In that case, as expected, the disease spreads out rapidly all over the country. In Figure S2 we present the number of sick people in each city as a function of time. Both models predict similar numbers of sick people and the spatial propagation can be easily seen. The cities are arranged from north to the south (top to bottom). As expected, the cities in the center, which are in strong relations with Tel-Aviv are infected earlier than the far north and south. Once the disease has arrived to a major city (Haifa in the north or Be'er Sheva in the south), spreading in the whole area is very rapid. The empty rows are the three industrial zones, where no population lives there.

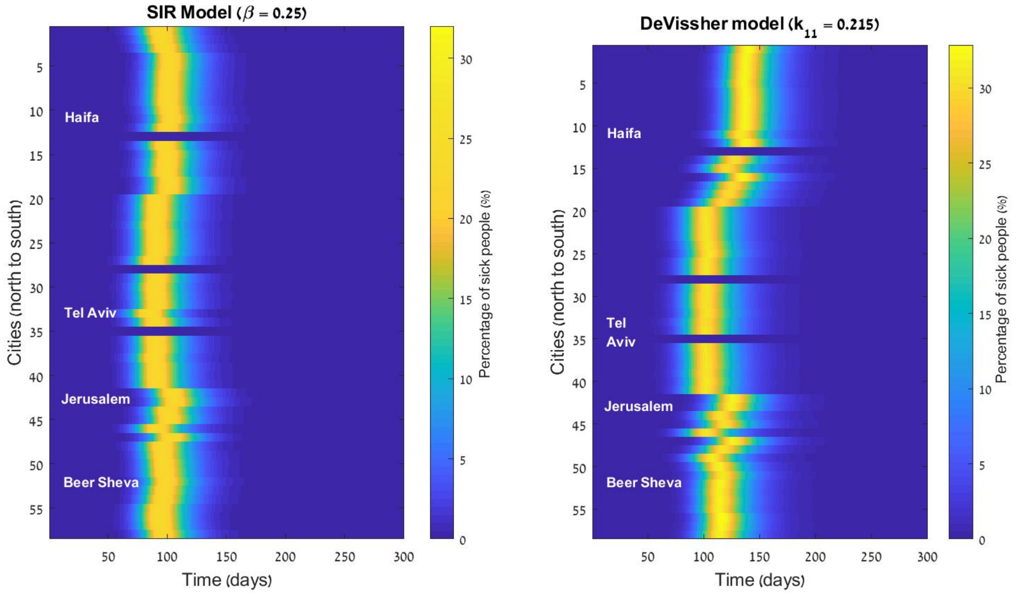

Figure S2. Percentage of sick in SIR model (left) and DeVisscher model (right). Each row represents the dynamics of one city, arranged from north to the south.

Population heterogeneity

Israel is a small country with roughly 10 million residents. It has been previously claimed that from an epidemiological point of view it can be considered as one large city[4]. It was shown that the major 12 cities in Israel exhibit similar temporal behavior, with the exception of the ultra-orthodox city of Bnei-Brak. This city demonstrated extremely unique dynamics in the current COVID-19 outbreak as well. It was also shown in that work that the cities which are distant from Tel-Aviv metropolitan, such as Haifa and Beer-Sheva, experienced later outbreaks. This geographical de-synchronization is consistent with our aforementioned results.

Data collected during the COVID-19 pandemic provides a unique opportunity for investigating the differences among a large ensemble of cities. Demographic, sociological, and cultural differences may lead to a variety of social behaviors[5]. The example of Bnei-Brak states that sociological variation may be translated into variation in epidemic dynamics. In Figure S3 we present results of the epidemiological model (EM) where all EM parameters values were the same for all cities. The initial condition is the number of sick people at April 3rd (the first day from which local data were available). Both standard transportation matrix or a weakened one (by a factor of 0.01) are presented. It is shown that both transportation matrices yield similar dynamics, but there is large variation among cities. Whereas some cities, like Ashdod and Jerusalem are fairly well fitted by a simple SIR model, other locations like Tel-Aviv or Ra'anana exhibit different dynamics. This demonstrates the need of tailoring the model, or at least its parameters, to the demographic and sociological characteristics of each particular city.

An exact evaluation of optimal parameters for each city is beyond the scope of this study. However, for the sake of demonstration, we have manually manipulated β - the infection rate value in the SIR model, to provide a better fit of the actual data. The results are presented in Figure S4. It is evident that the dynamics of the first 10 days fits the model predictions. In

some case we observe a sharp deviation after day 10, probably as a result of change in social behavior because of strengthening the social restrictions about ten days earlier. We see that even a coarse parameter adjustment may significantly improve the fitting quality and hopefully the accuracy of predictions. On a rapidly changing situation, there is no reason to use the same set of parameters for a period longer than approximately two weeks. We conclude that sociological variation should be taken into account in any EM and its parameters should be adapted on a local basis.

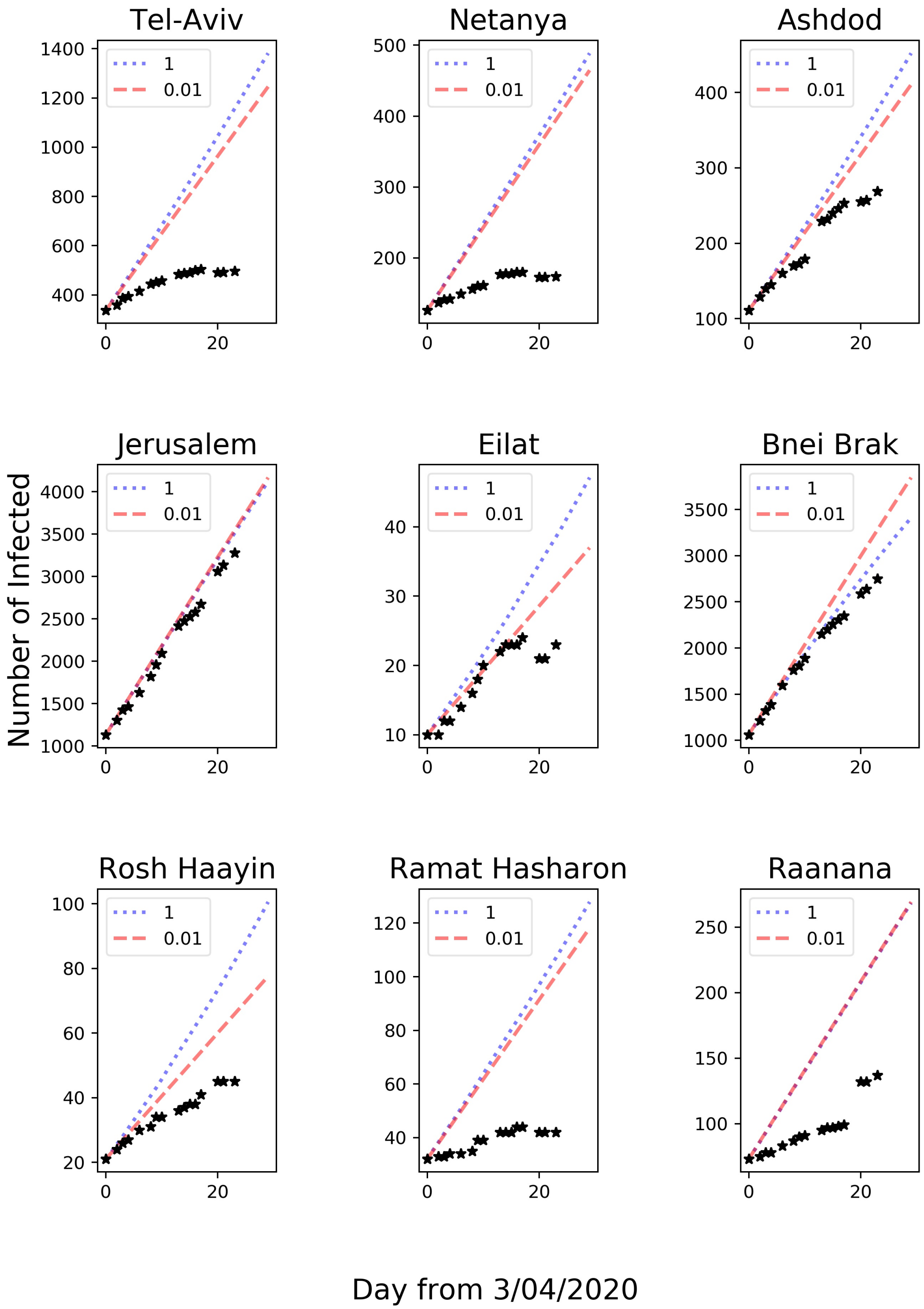

Figure S3. Number of sick people in a sample of cities, real data (∗) and results of SIR model with same parameters for all cities. Results of standard and reduced transportation matrix (by a factor 0.01)are presented.

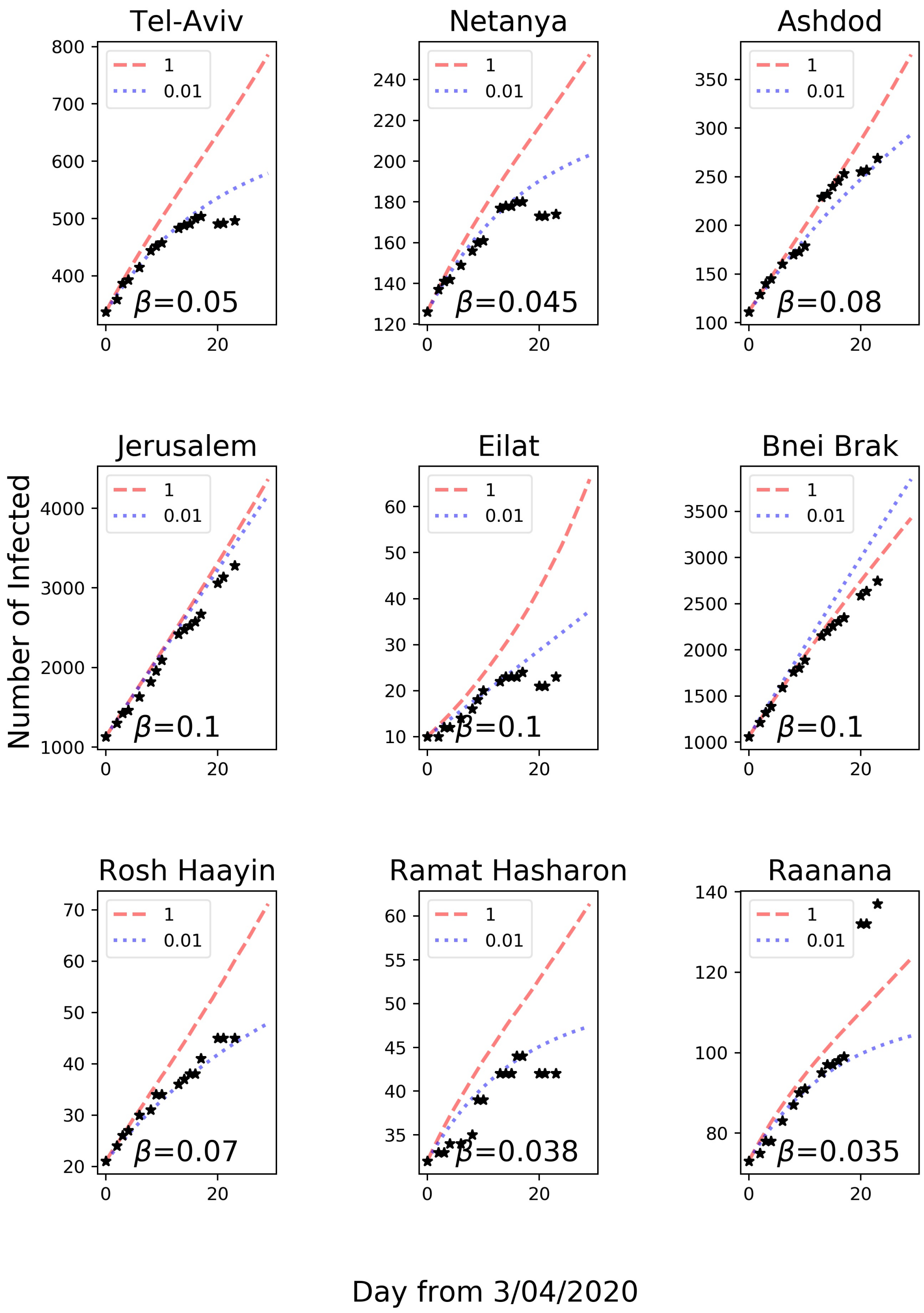

Figure S4. Number of sick people in a sample of cities, real data ($*$) and results of SIR model with specific parameter set for each city. Note that $R_0 = \beta/\gamma$ and in our model $\gamma = 0.1$ which means that taking $\beta = 0.15$ implies $R_0 = 1.5$. Results of standard and reduced transportation matrix (by a factor 0.01)are presented. Results of standard and reduced transportation matrix (by a factor 0.01)are presented.

References

1. Hunter,J. D., Matplotlib: A 2D Graphics Environment, *Computing in Science & Engineering*, 9 (3), 90-95, (2007)
2. Van Rossum, G., Drake Jr. FL., *Python reference manual*. Centrum voor Wiskunde en Informatica, Amsterdam (1995).
3. Israel Population and Immigration Authority - Residents in Israel by localities and age group (In Hebrew) https://data.gov.il/dataset/residents_in_israel_by_communities_and_age_groups/resource/64edd0ee-3d5d-43ce-8562-c336c24dbc1f (2020)
4. Barnea, O., Huppert, A., Katriel, G. and Stone, L., Spatio-temporal synchrony of influenza in cities across israel: The 'israel is one city' hypothesis. *PLOS ONE*, 9(3):1–11, 03 2014.
5. Poletti P., Ajelli M', Merler, S. The effect of risk perception on the 2009 H1N1 pandemic influenza dynamics. *PLoS One*, 6(2):e16460, (2011).
